# Plasma levels of progranulin and interleukin-6 in frontotemporal lobar degeneration

**DOI:** 10.1016/j.neurobiolaging.2014.10.023

**Published:** 2015-03

**Authors:** Linda Gibbons, Sara Rollinson, Jennifer C. Thompson, Andrew Robinson, Yvonne S. Davidson, Anna Richardson, David Neary, Stuart M. Pickering-Brown, Julie S. Snowden, David M.A. Mann

**Affiliations:** a Faculty of Human and Medical Sciences, Institute of Brain, Behaviour and Mental Health, University of Manchester, Manchester, UK; b Cerebral Function Unit, Department of Neurology, Greater Manchester Neuroscience Centre, Salford Royal Foundation Trust, Salford, UK

**Keywords:** Frontotemporal Lobar degeneration, Progranulin, Interleukin-6, Biomarkers

## Abstract

We have measured plasma progranulin and interleukin-6 in 230 patients with frontotemporal lobar degeneration (FTLD), 104 patients with Alzheimer's disease, and 161 control subjects. We have replicated previous findings of decreased levels of progranulin protein in FTLD because of mutations in *GRN* and show this is not observed in FTLD cases because of other causes. interleukin-6 levels were increased in FTLD overall, but these did not discriminate between clinical and genetic subtypes.

Frontotemporal lobar degeneration (FTLD) is the second most common cause of dementia in people younger than 65 years. The prototypical clinical syndromes are behavioral variant frontotemporal dementia, progressive nonfluent aphasia (PNFA), and semantic dementia (SD). Pathologically, about half of the cases show an accumulation of hyperphosphorylated tau proteins, some being associated with mutations in *MAPT*. Most others show an accumulation of the transactive DNA-binding protein, TDP-43, inherited cases being associated with mutations in progranulin (*GRN*) or hexanucleotide expansions in *C9ORF72*. However, when no gene mutation is known, it is not possible to accurately predict the underlying histology. A valid biological marker would not only help in formulating diagnosis, but could also serve as a potential index of treatment efficacy. Some progress has been made in this regard. Reductions in plasma levels of progranulin (PGRN) have been associated with *GRN* mutations (Finch et al., 2009, Ghidoni et al., 2008, Hsiung et al., 2011, Sleegers et al., 2009), and patients with FTLD have been reported to show increased cerebrospinal fluid or plasma levels of proinflammatory mediators (Sjögren et al., 2004, Galimberti et al., 2006, Bossù et al., 2011).

We therefore measured plasma PGRN and interleukin-6 (IL-6) in patients with FTLD, Alzheimer's disease (AD), and healthy (spouse) control subjects to ascertain whether levels of these have potential to discriminate patients with FTLD from other neurodegenerative disorders (AD) and healthy individuals, and also whether they can differentiate between clinical, neurohistologic, and genetic subtypes of FTLD. We investigated 230 patients with FTLD, 104 patients with AD, and 161 healthy control subjects (Table 1). Patients with FTLD fulfilled current clinical diagnostic criteria (Harris et al., 2013), and clinical diagnosis of AD was consistent with International Consensus Clinical Criteria (McKhann et al., 1984). Of patients with FTLD, 117 had behavioral variant frontotemporal dementia, 31 had FTD + MND, 35 had SD, 42 had PNFA, and 5 had progressive apraxia (PAX) (Table 1). Ten patients with FTLD bore mutations in *GRN*, one had a *MAPT* mutation, and 13 bore hexanucleotide repeat expansions in *C9ORF72*. The control subjects were spouses of the patients. They were neither related to each other nor to the patients (except through marriage). The 3 diagnostic groups did not differ as to mean age at onset or duration of illness when sampled. Within the FTLD group, age at onset was later in patients with FTD + MND and PNFA than those with FTD, and age when sampled was later in patients with PNFA than those with FTD. Duration of illness when sampled was longer in patients with SD than those with FTD + MND. There were no significant differences for age at onset, age when sampled, and duration of illness when sampled between patients with the *GRN* mutation, patients with the *C9ORF72* mutation and nonmutation bearers (see Table 1). Blood samples were obtained with informed consent and full ethical approval. Plasma was separated routinely and stored at −80 °C until required for assay. PGRN and IL-6 were assayed in triplicate by enzyme-linked immunosorbent assay using specific high sensitivity commercially available kits (DPGRNO kit, R&D Systems for PGRN, Quantikine H&S kit for IL-6) in accordance with the manufacturer's recommendations. The limits of detection were 0.17 ng/mL for PGRN and 0.20 pg/mL for IL-6. PGRN was measured on all subjects, whereas IL-6 measures were performed on all except 2 of the FTLD patients, all except 2 of the AD patients and all except 3 of the control subjects, where insufficient sample remained to complete both assays. DNA was extracted from white blood cells separated from whole blood by routine centrifugation. Screening for mutations in *MAPT*, *GRN*, and expansions in *C9ORF72* had already been performed on all subjects. Most of *GRN* rs5848 genotypes were obtained using Sequenom Mass array genotyping assays using 15 ng of DNA, as per the manufacturer's instructions. Additional genotypes for *GRN* rs5848 and *TMEM106B* rs1020004, rs6966915, and rs1990622 SNPs was obtained using Applied Biosystems assay numbers C__7452046_20, and C__7604953_10, C_31573289_10 and C__11171598_10, respectively. A total of 10 ng of each DNA was amplified and genotyped on the 7700 Real time PCR system. Genotype calls were made using SDS v2.3 software (Applied Biosystems, Foster City, CA). All statistical tests were performed using SPSS 16.0. Analysis of variance was used to compare age at onset, age at sampling, duration of illness, PGRN and IL-6 levels between the various clinical diagnostic and genetic groups with post hoc testing (subject to Bonferroni correction) when a significantly different analysis of variance result was obtained. Pearson correlation tests were used to test for correlation between IL-6 and PGRN measures, and between each of these and age at onset, age at sampling, and disease duration. Significance levels were set at *p* < 0.05.

Overall, there were no significant differences in mean plasma PGRN level between patients with FTLD, AD, or control subjects (Fig. 1A). However, mean plasma PGRN levels within the FTLD group were significantly lower in patients with *GRN* mutations than those with *C9ORF72* expansions, or nonmutation bearers (which in turn did not differ from each other) (Table 1; Fig. 1C). Hence, the range of values was 24.2–121.1 ng/mL (mean 53.7 ± 13.7 ng/mL) for control subjects, 27.4–93.9 ng/mL (mean 51.9 ± 12.6 ng/mL) for AD subjects and 23.7–85.0 ng/mL (52.7 ± 14.7 ng/mL) for FTLD subjects without *GRN* mutations. All FTLD patients with *GRN* mutations had plasma PGRN levels below 22.0 ng/mL (range 1.1–21.0 ng/mL, mean 11.1 ± 5.0 ng/mL), this being below the lower confidence interval for FTLD patients without *GRN* mutations. There were no significant differences in mean plasma PGRN levels according to clinical presentation (Fig. 1B). The mean plasma IL-6 level was significantly higher in FTLD than in AD and control subjects, the latter 2 groups not differing significantly (Fig. 1D). Hence, the range of values was −3.5 to 10.4 pg/mL (mean 3.5 ± 3.4 pg/mL) for control subjects, −1.0 to 9.6 pg/mL (mean 4.4 ± 2.7 pg/mL) for all FTLD subjects without *GRN* mutations, 2.0–5.6 pg/mL (mean 3.9 ± 0.9 pg/mL) for patients with *GRN* mutations and −2.2 to 7.4 pg/mL (mean 3.1 ± 2.5 pg/mL) for AD subjects. There were no significant differences in mean plasma IL-6 levels between the various FTLD clinical (Fig. 1E) or genetic (Fig. 1F) subgroups. There was a highly significant correlation between plasma PGRN and IL-6 levels in patients with FTLD, overall (*r* = 0.195, *p* = 0.003), but not so in AD, or control subjects. There were no significant correlations between plasma PGRN or IL-6 levels and age at onset in patients with FTLD (overall or stratified clinically or genetically), AD, or in control subjects, or between IL-6 levels and (current) duration of illness in patients with FTLD (separately or combined, clinically or genetically) or AD. However, there was a highly significant positive correlation (*r* = 0.466, *p* = 0.006) between PGRN levels and duration of illness for FTLD group overall, but not for any clinical or genetic subgroup, except for SD (*r* = 0.365, *p* = 0.037) and the nonmutational subgroups (*r* = 0.150, *p* = 0.034) where this also increased with duration of illness. We examined the effect of *GRN* allele status at rs5848 and *TMEM106B* variants rs1020004, rs1990622, and rs6966915 on PGRN or IL-6 plasma levels in control subjects (n = 133), AD (n = 82), and FTLD cases, excluding mutations in *MAPT*, *GRN*, and *C9ORF72* (n = 184), but no significant correlations were seen either for FTLD overall or when stratified according to clinical diagnostic or genetic subgroup (see online Supplementary Tables 1–4).

The results of the present study clearly demonstrate that blood levels of PGRN are greatly reduced in patients bearing *GRN* mutations compared with others with FTLD, those with AD, or control subjects, and therefore support previous findings (Finch et al., 2009; Ghidoni et al., 2009; Hsiung et al., 2011, Sleegers et al., 2009). Such observations add to the argument that measurement of plasma PGRN can act as a relatively quick and accurate surrogate to screen for patients bearing mutations in *GRN*. Interestingly, the *GRN* group contained a single patient with Q415X “mutation,” which had not been previously shown to segregate with disease or to be pathogenic in functional assay (Baker et al., 2006). However, present data showing low PGRN levels in the patient with this mutation (8.0 ng/mL) would imply the mutation is indeed pathogenic. Although mean IL-6 levels are higher in FTLD than in AD and control subjects, the differences are not sufficiently clear cut as to provide a useful biomarker for FTLD either overall or according to clinical or genetic subtype. Previously, Bossù et al. (2011) reported patients with *GRN* mutations showed significantly elevated IL-6 levels compared with FTLD patients without such mutations. However, we find that levels in patients with *GRN* mutations are not significantly elevated. Differences in *GRN* mutation, patient variability and small sample sizes giving rise to chance findings are likely responsible for such inconsistent findings. We find a highly significant correlation between plasma PGRN and IL-6 levels in patients with FTLD, but not in AD or control subjects. This finding in FTLD is perhaps not surprising given that PGRN expression is driven by IL-6 secretion (Frampton et al., 2012), perhaps reflecting increased microglial activity especially in *GRN* carriers (Ahmed et al., 2007, Cagnin et al., 2004) or in normal control subjects. However, if so, it is curious as to why a similar association is not seen in AD where senile plaques are heavily invested with microglial cells.

## Disclosure statement

The authors disclose no actual or potential conflicts of interest.

## Figures and Tables

**Fig. 1:**
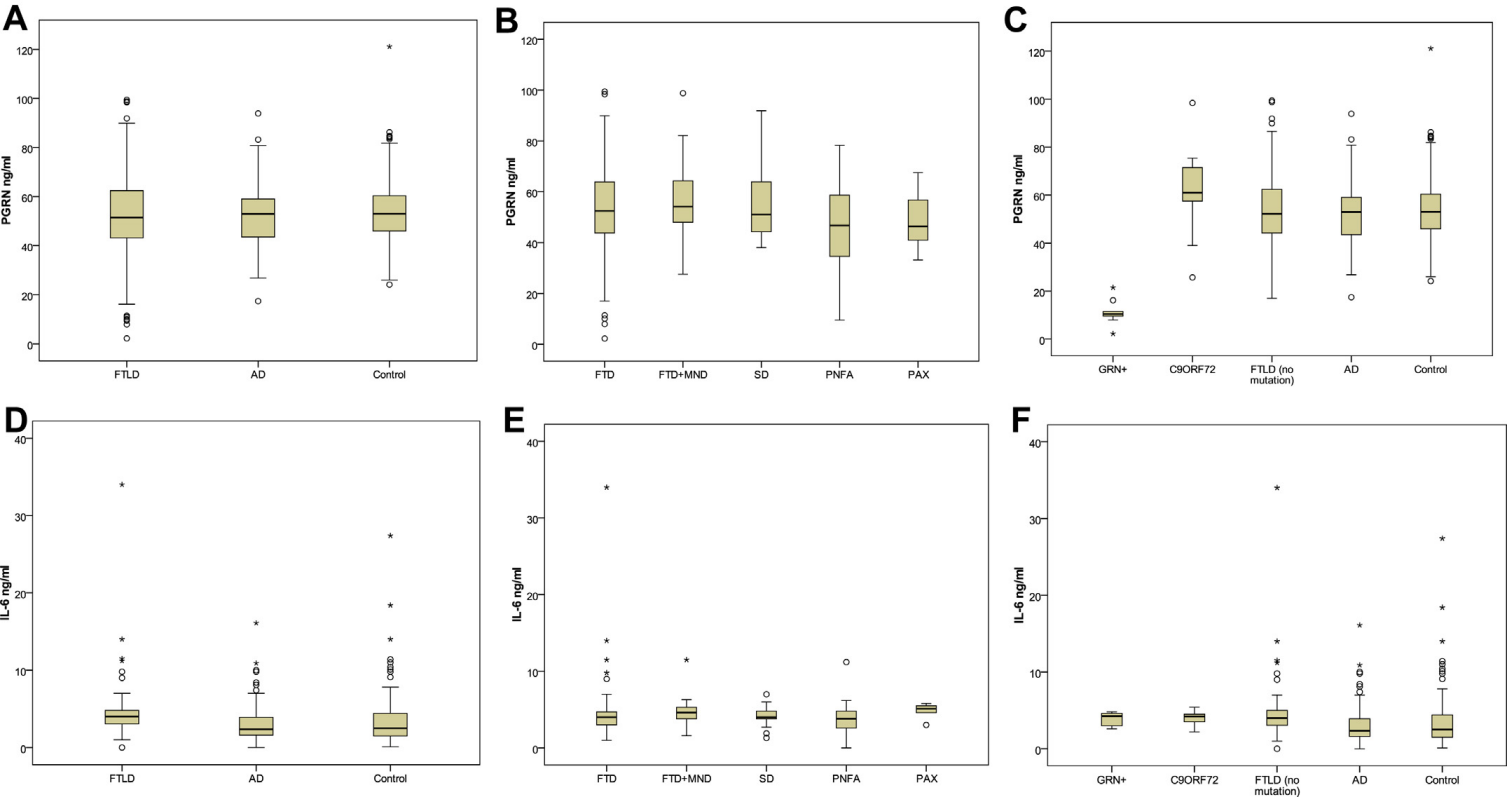
Boxplots for measures of plasma PGRN (A-C) and IL-6 (D-F) levels for Frontotemporal Lobar degeneration (FTLD), Alzheimer's Disease (AD) and control groups, collectively, (A,C), and for FTLD groups stratified by clinical presentation into those with Frontotemporal dementia (FTD), FTD with Motor Neurone Disease (FTD+MND), Semantic dementia (SD), Progressive non-fluent Aphasia (PNFA) and Progressive Apraxia (PAX) (B,E), and by genetics into cases with GRN mutations, expansions in C9ORF72 or no known mutation (C,F).

**Table 1:** Mean (±SD) age at onset, age when sampled, current duration of illness, PGRN and IL-6 levels for patients with FTLD, both overall and when stratified according to clinical or genetic status, AD, and control subjects

Diagnostic group	Age at onset (y)	Age when sampled (y)	Duration (y)	PGRN (ng/mL)	IL-6 (pg/mL)
bvFTD (n = 117)	58.6 ± 8.9	62.5 ± 8.8	4.0 ± 3.0	54.7 ± 15.7	4.4 ± 3.4∗∗∗∗
bvFTD + MND (n = 31)	64.4 ± 8.2∗	67.2 ± 8.3	2.7 ± 1.5	56.6 ± 15.7	4.7 ± 1.8
SD (n = 35)	60.5 ± 6.0	65.7 ± 6.8	5.0 ± 3.0∗∗	55.5 ± 13.6	4.3 ± 1.1
PNFA (n = 42)	65.2 ± 7.0∗	68.6 ± 7.1∗	3.4 ± 2.1	49.6 ± 13.4	3.8 ± 1.9
PAX (n = 5)	66.2 ± 14.7	69.7 ± 14.2	3.5 ± 1.9	49.0 ± 13.5	4.8 ± 1.1
All FTLD (n = 230)	61.1 ± 8.7	64.9 ± 8.7	3.9 ± 2.7	52.1 ± 17.1	4.3 ± 2.6
*MAPT*+ (n = 1)	58	58.9	0.9	39.2	1.9
*GRN*+ (n = 10)	59.8 ± 4.0	62.1 ± 4.7	2.4 ± 1.0	11.1 ± 5.0∗∗∗	3.9 ± 0.9
*C9ORF72* + (n = 13)	59.0 ± 8.2	62.7 ± 10.0	4.6 ± 4.8	61.0 ± 18.0	4.0 ± 0.9
Nonmutation bearers (n = 206)	61.2 ± 8.9	65.2 ± 8.7	3.9 ± 2.6	53.5 ± 14.6	4.3 ± 2.8
AD (n = 104)	62.3 ± 8.3	66.2 ± 8.1	3.7 ± 1.8	51.9 ± 12.6	3.1 ± 2.5
Controls (n = 161)	NA	65.1 ± 9.4	NA	53.7 ± 13.7	3.5 ± 3.4

Key: bvFTD, behavioral variant frontotemporal dementia; FTLD, frontotemporal lobar degeneration; IL-6, interleukin-6; NA, not applicable; PGRN, progranulin; SD, semantic dementia.

∗ *p* < 0.006 compared with bvFTD.

∗∗ *p* < 0.01 compared with bvFTD + MND.

∗∗∗ *p* < 0.001 compared with C9ORF72+ and nonmutation bearers.

∗∗∗∗ *p* < 0.018 and 0.001 compared with AD and controls, respectively.
