# Supplementary material for: Plasma levels of progranulin and interleukin-6 in frontotemporal lobar degeneration
Source: Neurobiol Aging. 2015 Mar;36(3):1603.e1–4. doi: 10.1016/j.neurobiolaging.2014.10.023 (PMC4504979; doi:10.1016/j.neurobiolaging.2014.10.023)
Supplement: Supplementary Tables 1–4 [file mmc1.docx]

| Group | Mean PGRN level | N within each group  (CC, CT, TT) | PGRN level | | |
| --- | --- | --- | --- | --- | --- |
|  |  |  | CC | CT | TT |
| AD (n=82) | 51.5±12.3 | 47, 32, 3 | 52.3±12.01 | 50.9±12.88 | 45.7±11.59 |
| Controls (n=133) | 53.6±14.0 | 77, 45, 11 | 55.4±14.86 | 50.9±13.12 | 51.6±9.58 |
| FTLD (n=184) | 53.6±14.9 | 109, 60, 15 | 55.4±14.59 | 50.2±14.58 | 54.4±16.71 |
| FTD (n=91) | 54.2±15.5 | 57, 26, 8 | 55.5±15.95 | 52.3±15.70 | 51.5±12.42 |
| FTD/MND (n=26) | 56.4±15.1 | 10, 15, 1 | 54.1±10.07 | 55.1±14.41 | 98.8 |
| SD (n=31) | 55.3±13.9 | 22, 6, 3 | 58.0±15.39 | 46.9±5.10 | 52.2±5.43 |
| PNFA/PAX (n=36) | 48.7±13.2 | 20, 13, 3 | 53.1±11.63 | 41.8±12.55 | 49.9±18.13 |

Supplementary Table S1 Mean (±SD) PGRN level in AD, controls and FTLD patients, overall and stratified according to rs5848 genotype.

| Group | Mean IL-6 level | N within each group  (CC, CT, TT) | IL-6 level | | | P |
| --- | --- | --- | --- | --- | --- | --- |
|  |  |  | CC | CT | TT |  |
| AD (n=81) | 3.2±2.4 | 46, 32, 3 | 3.1±2.2 | 3.2±2.7 | 4.0±1.5 | 0.819 |
| Controls (n=130) | 3.5±3.5 | 98, 24 , 8 | 3.3±3.1 | 4.4±4.5 | 2.7±1.6 | 0.981 |
| FTLD (n=183) | 4.4±2.9 | 109, 59, 15 | 4.4±3.3 | 4.5±1.9 | 4.0±2.4 | 0.624 |
| FTD (n=90) | 4.5±3.7 | 57, 25, 8 | 4.6±4.3 | 4.8±2.5 | 3.5±1.6 | 0.688 |
| FTD/MND (n=26) | 4.7±1.9 | 10, 15, 1 | 5.3±2.5 | 4.3±1.4 | 4.0 | 0.426 |
| SD (n=31) | 4.3±1.0 | 22, 6, 3 | 4.3±0.8 | 4.5±1.4 | 3.5±1.6 | 0.366 |
| PNFA/PAX (n=36) | 4.0±1.9 | 20, 13, 3 | 3.7±1.6 | 4.1±1.1 | 5.9±4.8 | 0.159 |

Supplementary Table S2 Mean (±SD) IL-6 level in AD, controls and FTLD patients, overall and stratified according to rs5848 genotype.

| Group | Mean PGRN level | N within each group  (TT, TC, CC) | Mean PGRN Level | Mean PGRN Level | Mean PGRN Level | P value |
| --- | --- | --- | --- | --- | --- | --- |
| rs1020004 |  |  | TT | TC | CC |  |
| ALL (n=164) | 53.8±14.8 | 76,74,14 | 54.1±14.9 | 53.6±15.2 | 53.7±13.2 | 0.904 |
| FTD (n=83) | 53.9±15.4 | 32, 44, 7 | 53.8±14.2 | 54.6±16.5 | 50.4±14.9 | 0.805 |
| FTD/MND (n=23) | 57.6±14.8 | 15, 7, 1 | 57.5±17.3 | 56.9±9.6 | 64.1 | 0.910 |
| SD (n=28) | 55.8±14.4 | 14, 11, 3 | 59.5±14.3 | 53.3±14.9 | 55.8±11.3 | 0.336 |
| PNFA/PAX (n=30) | 48.9±12.9 | 15, 12, 3 | 46.4±12.4 | 48.2±12.7 | 64.1±6.0 | 0.086 |
|  |  |  |  |  |  |  |
| rs1990622 |  | AA, GA, GG | AA | GA | GG |  |
| ALL FTLD (n=109) | 55.4±15.0 | 33,61,16 | 55.3±17.0 | 54.8±15.2 | 54.5±9.8 | 0.825 |
| FTD (n=55) | 56.7±15.0 | 12, 35, 8 | 58.4±13.2 | 56.3±16.7 | 55.6±9.2 | 0.854 |
| FTD/MND (n=19) | 57.8±15.9 | 9, 7, 3 | 61.8±20.4 | 54.5±8.7 | 53.7±15.9 | 0.614 |
| SD (n=16) | 55.9±15.3 | 5, 9, 2 | 56.3±16.1 | 55.0±17.4 | 59.1±1.1 | 0.947 |
| PNFA/PAX (n=19) | 50.1±14.0 | 7, 9, 3 | 41.2±13.7 | 52.1±11.4 | 65.1±6.4 | 0.027 |
|  |  |  |  |  |  |  |
| rs6966915 |  | GG, GA, AA | GG | GA | AA |  |
| ALL FTLD (n=98) | 55.8±15.5 | 34,50,15 | 57.2±16.8 | 55.8±16.1 | 55.3±10.0 | 0.923 |
| FTD (n=49) | 57.9±15.4 | 12, 29, 8 | 58.4±13.2 | 58.3±17.6 | 55.6±9.2 | 0.929 |
| FTD/MND (n=16) | 58.2±17.3 | 9, 5, 2 | 61.8±20.4 | 55.2±10.6 | 49.7±20.3 | 0.637 |
| SD (n=14) | 55.2±15.4 | 5, 7, 2 | 56.3±16.1 | 53.3±18.3 | 59.1±1.1 | 0.894 |
| PNFA/PAX (n=19) | 50.5±14.0 | 8,8,3 | 42.7±13.5 | 52.8±12.0 | 65.1±6.4 | 0.039 |

Supplementary Table S3 Mean (±SD) PGRN level in FTLD patients, overall and according to clinical diagnostic groups, stratified according to *TMEM106B* genotype.

| Group | Mean IL-6 level | N within each group  (TT, TC, CC) | Mean IL-6 Level | Mean IL-6 Level | Mean IL-6 Level | P value |
| --- | --- | --- | --- | --- | --- | --- |
| rs1020004 |  |  | TT | TC | CC |  |
| FTLD (n=164) | 4.4±2.99 | 76, 74, 14 | 4.2±1.8 | 4.7±4.0 | 4.7±2.2 | 0.705 |
| FTD (n=82) | 4.5±3.87 | 31, 44, 7 | 4.0±2.0 | 4.9±5.0 | 4.2±0.8 | 0.590 |
| FTD/MND (n=23) | 4.8±1.90 | 15, 7, 1 | 4.9±2.2 | 4.6±1.2 | 5.9 | 0.813 |
| SD (n=28) | 4.3±1.07 | 14, 11, 3 | 4.3±1.2 | 4.3±1.0 | 4.3±1.1 | 0.968 |
| PNFA/PAX (n=30) | 4.1±2.01 | 15, 12, 3 | 3.7±1.2 | 4.1±1.8 | 6.1±4.8 | 0.168 |
|  |  |  |  |  |  |  |
| rs1990622 |  | AA, GA, GG | AA | GA | GG |  |
| FTLD (n=109) | 4.5±3.42 | 32, 61, 16 | 3.7±1.6 | 4.7±4.2 | 5.5±2.4 | 0.564 |
| FTD (n=54) | 4.5±4.42 | 11, 35, 5 | 2.9±1.5 | 5.0±5.3 | 4.4±1.0 | 0.364 |
| FTD/MND (n=19) | 4.9±2.10 | 9, 7, 3 | 4.3±1.5 | 4.7±1.4 | 7.1±3.9 | 0.116 |
| SD (n=16) | 4.4±1.24 | 5, 9, 2 | 4.9±1.6 | 4.0±1.1 | 5.1±0.9 | 0.346 |
| PNFA/PAX (n=19) | 4.4±2.40 | 7, 9, 3 | 3.4±1.4 | 4.1±2.1 | 7.2±3.4 | 0.057 |
|  |  |  |  |  |  |  |
| rs6966915 |  | GG, GA, AA | GG | GA | AA |  |
| FTLD (n=97) | 4.5±3.51 | 32, 50, 15 | 3.7±1.6 | 4.8±4.6 | 5.1±1.9 | 0.788 |
| FTD (n=48) | 4.5±4.66 | 11, 29, 8 | 2.9±1.5 | 5.1±5.8 | 4.4±1.0 | 0.384 |
| FTD/MND (n=16) | 4.5±1.44 | 9, 5, 2 | 4.3±1.5 | 4.7±1.6 | 5.0±1.3 | 0.786 |
| SD (n=14) | 4.7±1.10 | 5, 7, 2 | 4.9±1.6 | 4.3±0.8 | 5.1±0.9 | 0.607 |
| PNFA/PAX (n=19) | 4.2±2.40 | 8, 8, 3 | 3.4±1.3 | 3.9±2.2 | 7.2±3.4 | 0.046 |

Supplementary Table S4 Mean (±SD) IL-6 level in FTLD patients, overall and according to clinical diagnostic groups, stratified according to *TMEM106B* genotype.
